# Correction: Comparison of commercially available, rapid, point-of-care C-reactive protein assays among children with febrile illness in southwestern Uganda

**DOI:** 10.1371/journal.pgph.0006247

**Published:** 2026-04-02

**Authors:** Caitlin A. Cassidy, Lydiah Kabugho, Georget Kibaba, Bradley Lin, Brandon Hollingsworth, Emmanuel Baguma, Jonathan J. Juliano, Edgar M. Mulogo, Ross M. Boyce, Emily J. Ciccone

In the Malaria and CRP subsection of the Results, there is an error in the last sentence of the paragraph. The correct sentence is: The mean CRP (SD) as measured using the Afinion assay in malaria positive and malaria negative patients was 58.9 (8.8) mg/L and 20.5 (2.8) mg/L, respectively.
