# Supplementary material for: Comparison of commercially available, rapid, point-of-care C-reactive protein assays among children with febrile illness in southwestern Uganda
Source: PLOS Glob Public Health. 2024 Jan 19;4(1):e0002727. doi: 10.1371/journal.pgph.0002727 (PMC10798459; doi:10.1371/journal.pgph.0002727)
Supplement: S1 Text — (PDF) [file pgph.0002727.s001.pdf]

Version: OCT 2020– STAR STUDY

Study ID (Site **-** Day - Month - Number): BHC --- --- ---

SECTION 1: DEMOGRAPHIC INFORMATION

**Patient:**	**Sex:**<br>☐ Male<br>☐ Female	**Age:**	**Home Location:**
		**Village/LC1:**	**Parish/Ward:**

Consent Obtained: ☐ Yes ☐ No (Patient excluded, form complete)

SECTION 2: CLINICAL INFORMATION

Symptoms: ☐ fever

☐ skin rash ☐ muscle aches

☐ cough ☐ fatigue

☐ headache ☐ sore throat

☐ runny nose ☐ joint pains

☐ diarrhea ☐ vomiting

☐ not eating ☐ fast breathing

☐ convulsions ☐ bleeding

☐ coma ☐ chest in-drawing

☐ Other: ____________________

Clinical History:

Days since first fever: _______

Seen at another clinic for this same problem?
☐ Yes
☐ No

Have you taken drugs for malaria in the last two weeks?
☐ Yes
☐ No

Have you taken drugs to treat infection (antibiotics) in the last two weeks?
☐ Yes
☐ No

Past Medical History:

☐ Asthma
☐ Diabetes
☐ Epilepsy
☐ Heart problems
☐ High blood pressure
☐ ISS
☐ Malaria
☐ Tuberculosis

Other conditions:

SECTION 3: OBJECTIVE DATA

Temperature	RR	Heart Rate	Oxygen (%)	Weight (kg)	**Malaria Test Result:** ☐ POS ☐ NEG

SECTION 4: CONFIRM INCLUSION CRITERIA

**Does patient have:**	☐ History of fever in last 7 days	*If the patient meets this criterion, then they are eligible for further testing.* ***Please perform the tests below:***

SECTION 5: RESULTS OF CRP TESTS

Actim CRP:	Afinion CRP
BTNX CRP:	SD Biosensor Malaria/CRP Duo:

SECTION 6: PATIENT TREATMENT & DISPOSITION

**Treatment**	☐ Antibiotics: ______________________________	☐ Other: ______________________________	
**Disposition**	☐ Discharged from OPD	☐ Admitted to IPD	☐ Referred
	☐ Other (specify):		
