# Supplementary material for: Comparison of commercially available, rapid, point-of-care C-reactive protein assays among children with febrile illness in southwestern Uganda
Source: PLOS Glob Public Health. 2024 Jan 19;4(1):e0002727. doi: 10.1371/journal.pgph.0002727 (PMC10798459; doi:10.1371/journal.pgph.0002727)
Supplement: Attachment — Submitted filename: Ciccone_ResponseToReviewers_(14OCT23).docx [file pgph.0002727.s003.docx]

October 24, 2023

Jianhong Zhou, PhD
Staff Editor
PLOS Global Public Health

Dear Dr. Zhou,

Thank you for the constructive review of our manuscript entitled, “Comparison of commercially available, rapid, point-of-care C-reactive protein assays among children with febrile illness in southwestern Uganda” (PGPH-D-23-00899). Please find our responses (in blue) to reviewers’ comments below.

**Journal Requirements**

Comment 1: Please include a complete copy of PLOS’ questionnaire on inclusivity in global research in your revised manuscript.

*Response: We have included this questionnaire with our revised manuscript.*

Comment 2: We noticed that you used "data not shown" in the manuscript. We do not allow these references, as the PLOS data access policy requires that all data be either published with the manuscript or made available in a publicly accessible database. Please amend the supplementary material to include the referenced data or remove the references.

*Response: We intended to use “data not shown” to state that the referenced data were not in table 2, but this reference is unneeded. We have removed the reference from the manuscript.*

Comment 3: We have noticed that you have a list of Supporting Information legends in your manuscript. However, there are no corresponding files uploaded to the submission. Please upload them as separate files with the item type 'Supporting Information'.

*Response: We have now included the referenced case report form as part of the supporting information.*

**Reviewer #1**

General comments: This manuscript describes a study that evaluated the analytical performance of 3 RDT tests for CRP. The evaluation was performed using samples from 150 children presenting with febrile symptoms. The authors reported that the tests showed acceptable performance compared with the reference test. The study is well designed and clear, but it would be useful if the authors could provide more information or explanation as follows.

Comment 1: The author does not explain why these three specific tests were selected rather than others. It would be helpful to understand the reasons behind this choice.

*Response:* *To identify C-reactive protein tests for this study, we reviewed the existing studies of CRP measurement in resource-limited settings, discussing test options with one of the research groups performing studies of CRP testing for febrile illness (1,2), and conducted a broad internet search. We then made final selections based on the following criteria. First, all of the tests needed to be commercially available (as opposed to in-house assays developed by individual laboratories). Second, for the point-of-care tests, they needed to be simple enough to be performed by personnel with limited training, not require electricity, and provide at least semi-quantitative results that included cut-offs of 40 and 80mg/L. Third, for the lab-based assay, it had to be implementable in a peripheral health facility in Uganda with a basic lab (i.e. using a small analyzer as opposed to a large high-throughput system), have published data on its performance, and be available through a distributor in Uganda who could provide training to study lab technicians. Fourth, all tests had to be CE marked. Finally, cost was taken into consideration; we selected the assays that had a lower cost per test. In the text, we now include the sentence “These CRP tests were selected because of their feasibility for implementation in a resource-limited facility, lower costs, and because they are CE marked.” (lines 102-103).*

Comment 2: Could the author provide more details about the testing procedure? Were all analyses (RDTs and reference standards) performed by the same laboratory technician? Was the technician blinded to the results of Afinion (reference standard)? If not, this could be a limitation of the study and should be mentioned in the discussion.

*Response: There were three laboratory technicians who alternated conducting the study tests while others continued with the routine clinical work of the laboratory. Due to budget constraints and to avoid impeding the routine clinical work, one technician conducted all the tests for each individual participant. Therefore, they were not blinded to the results of the reference standard. We have clarified this in the methods section (lines 112-114). We also have added this as a limitation to the study in the discussion section (line 281).*

Comment 3: The author could discuss better the complexity of having a "universal" threshold. The author should explain why he/she decided to analyze 40 and 80 as cut-offs (why not include 20 to have a better comparison between the 3 RDTs)?

*Response: We have expanded our discussion of the complexity of threshold choice and subsequent assay selection in the discussion (lines 249-252). As mentioned in the Data Analysis section of the Methods, the cut-offs of 40 and 80mg/L were chosen to be consistent with previously published studies of the use of CRP in the context of acute respiratory illness. Therefore, we were primarily interested in the performance of the Actim and BTNX tests because the possible results for those semi-quantitative tests (<10, 10-40, 40-80, and >80 mg/L) aligned with these cut-offs of interest. They are semi-quantitative and provide more information than the binary Duo test. We could not compare the three RDTs at a threshold of 20 mg/L as this was not a possible result for the Actim and BTNX tests.*

Comment 4: Are N=50 samples sufficient to make statements about the performance of the Duo RDT?

*Response: The reviewer makes a strong point that the results of the Duo RDT may be limited by the lower sample size. We have updated the discussion (line 240-241) to reflect this “The Duo CRP test perfectly categorized those with CRP <20 mg/L, though we tested fewer samples with Duo (n=50) than with Actim and BTNX (n=150) and mismeasurement may have been greater in the larger samples.” However, our sample size of 50 is above the minimum sample size (n=30) for ensuring sufficient precision for Kappa estimates that has been proposed in the literature.(3)*

Comment 5: The caption of Figure 1 (line 190) should include more details about the analysis performed.

*Response: We agree with the reviewer that the caption did not contain sufficient detail. We have updated the caption to reflect the meaning of the forest plot: “Sensitivity and specificity (diamonds) and corresponding 95% confidence intervals (lines) of Actim and BTNX RDTs at cut-offs of 40 and 80 mg/L with Afinion as the reference standard. P-values shown are for overall Wald test assessing whether sensitivities and specificities differ.” Additionally, we have updated the methods (line 135) to describe the confidence interval methods: “The sensitivity and specificity, with corresponding 95% CIs based on the modification of the Wilson interval developed by Yu et. al., of the Actim and BTNX tests were calculated using Afinion as the reference test”.*

Comment 6: Row 186, typo "performance".

*Response: Corrected.*

**Reviewer #2**

General comments: In this paper, the authors compare three semi-quantitative CRP RDTs to Afinion as goal standard. With the inappropriate use of antibiotics in LMICs due to the lack of appropriate and practical diagnostic tool to discriminate bacterial infections to other infections, CRP can be an alternative to support this diagnostic approach. The semi-quantitative CRP is easy to use by non-technician and can be perform outside conventional lab. Nevertheless, there is a need to assess the performance of these semi-quantitative CRP. I found this paper interesting and it is a great pleasure to read this paper.

Comment 1: Just one point that the authors can mention is the production of CRP in malaria infected people. This can make the implementation difficult in malaria endemic area, if an approach with positivity cut-offs are not determined for bacterial infections in these areas.
Except that point, I think this paper can be published.

*Response: We thank the reviewer for this excellent point. Indeed, as we found in our study, CRP on average is higher in individuals with evidence of malaria infection. While this was not the primary focus of our paper, we have included a brief paragraph in the discussion (lines 264-272) addressing this important issue.*

Kind regards,

Emily J. Ciccone, MD, MHS

Assistant Professor of Medicine

Division of Infectious Diseases

University of North Carolina School of Medicine

**References**

1.	Keitel K, Kagoro F, Samaka J, Masimba J, Said Z, Temba H, et al. A novel electronic algorithm using host biomarker point-of-care tests for the management of febrile illnesses in Tanzanian children (e-POCT): A randomized, controlled non-inferiority trial. Tumwine JK, editor. PLOS Medicine. 2017 Oct 23;14(10):e1002411.

2.	Keitel K, Samaka J, Masimba J, Temba H, Said Z, Kagoro F, et al. Safety and Efficacy of C-reactive Protein–guided Antibiotic Use to Treat Acute Respiratory Infections in Tanzanian Children: A Planned Subgroup Analysis of a Randomized Controlled Noninferiority Trial Evaluating a Novel Electronic Clinical Decision Algorithm (ePOCT). Clinical infectious diseases. 2019;69(11):1926–34.

3.	McHugh ML. Interrater reliability: the kappa statistic. Biochem Med (Zagreb). 2012 Oct 15;22(3):276–82.
